# Associations between eating meals, watching TV while eating meals and weight status among children, ages 10–12 years in eight European countries: the ENERGY cross-sectional study

**DOI:** 10.1186/1479-5868-10-58

**Published:** 2013-05-15

**Authors:** Frøydis N Vik, Helga Birgit Bjørnarå, Nina C Øverby, Nanna Lien, Odysseas Androutsos, Lea Maes, Natasa Jan, Eva Kovacs, Luis A Moreno, Alain Dössegger, Yannis Manios, Johannes Brug, Elling Bere

**Affiliations:** 1 Department of Public Health, Sport and Nutrition, University of Agder, Postboks 422, Kristiansand N-4604, Norway; 2 Department of Nutrition, University of Oslo, Oslo, Norway; 3 Department of Nutrition and Dietetics, Harokopio University, Athens, Greece; 4 Department of Public Health, Ghent University, Ghent, Belgium; 5 Slovenian Heart Foundation, Ljubljana, Slovenia; 6 Department of Pediatrics, Pecs University, Pecs, Hungary; 7 GENUD (Growth, Exercise, Nutrition and Development) research group. Facultad de Ciencias de la Salud, Universidad de Zaragoza, Zaragoza, Spain; 8 Federal Office of Sport, Magglingen, Switzerland; 9 EMGO Institute for Health and Care Research and the department of Epidemiology and Biostatistics, VU University Medical Center, Amsterdam, the Netherlands

**Keywords:** Children, Weight status, Meals, Breakfast, TV viewing, Never TV at meals

## Abstract

**Background:**

To assess the association of eating meals, and never watching TV while eating meals, with weight status among children, ages 10–12 years across Europe.

**Methods:**

7915 children (mean age: 11.5 years) in eight European countries (Belgium, Greece, Hungary, the Netherlands, Norway, Slovenia, Spain and Switzerland) completed a questionnaire at school. Data on meals eaten the day before questionnaire administration and the frequency of eating meals while watching TV were collected. Height and weight of the children were objectively assessed. Multinomial and binary regression analyses were conducted to test associations of eating meals (adjusted for gender and ethnicity) and never watching TV while eating meals (adjusted for gender, ethnicity and total TV time) with overweight/obesity, and to test for country- and socio-demographic differences.

**Results:**

The proportions of children reporting eating breakfast, lunch and dinner were 85%, 96%, and 93% respectively, and 55%, 46% and 32% reported to never watch TV at breakfast, lunch and dinner respectively. The children who ate breakfast (OR = 0.6 (95% CI 0.5-0.7)) and dinner (OR = 0.4 (95% CI 0.3-0.5)), had lower odds of being overweight compared to those who did not. The children who never watched TV at lunch (OR = 0.7 (95% CI 0.7-0.8)) and dinner (OR = 0.8 (95% CI 0.7-0.9)) had lower odds of being overweight compared to those who watched TV at the respective meals.

**Conclusions:**

The odds of being overweight was lower for children who ate breakfast and dinner compared to those who did not eat the respective meals. The odds of being overweight was lower for children who reported to never watch TV at lunch and dinner compared to those who did. A focus towards meal frequency and watching TV during meals in longitudinal and interventions studies in prevention of overweight and obesity, may contribute to a better understanding of causality.

## Background

There is convincing evidence for an increase in overweight and obesity among children and adolescents across Europe and beyond over the past decades [1-3]. Overweight and obesity are the results of a positive energy balance over time [4]. Childhood overweight may track into adulthood [5], therefore it is of great importance to promote obesity prevention at an early stage.

One of the potential energy-balance related behaviors (EBRB’s) is eating regular meals [6]. Main meals are often defined as eating breakfast, lunch and dinner [7]. Researchers have regarded skipping breakfast as a behavior associated with the risk of becoming overweight during adolescence [8,9]. Eating breakfast on a regular basis together with a regular meal pattern have in cross-sectional studies been shown to be associated with lower risk for overweight and obesity among children and adolescents [10-12]. Several observational studies described in a review showed a consistent cross-sectional association of skipping meals with an increased obesity risk in children, but not a longitudinal association [13]. Also a possible protective effect of an increased daily meal frequency on obesity in children has been reported [11,12].

It has been well established that high levels of sedentary behavior are associated with an increased risk of weight gain [14,15] and metabolic disease [16] - but the evidence among children is certainly not conclusive [17]. Most research is related to television (TV) viewing [18,19] and other screen time activities [20,21], i.e. specific sedentary activities that only explain very little of total variance in total accelerometer assessed sedentary time [22]. Several potential mechanisms have been proposed to explain the relationship between TV watching and obesity, including increased energy intake [23], reduced time available for physical activity [24], increased sedentary behavior [25] and reduced resting metabolic rate [26] (for which there is little supporting evidence [27]). Recently, there has been more attention towards the influence of TV advertising of food and beverages that targets children as an important driver of childhood and adolescent obesity [28]. Thus, the relationship between TV watching and obesity may not only be due to the sedentary activity as such, but may also be partly associated with eating behaviors in front of the TV. Observational studies have reported positive associations between prevalence of TV viewing during meals both with higher mean BMI and poorer dietary quality [29,30]. Both watching TV and eating while watching TV have been found to be positively and independently associated with overweight [30], suggesting that both sedentary behaviors from time spent watching TV as well as eating while watching TV contribute to overweight in children [30]. There might also be a link between high TV viewing and an unhealthy lifestyle in general [31]. The relationship between the main meals (and not only breakfast) and overweight and obesity, and also if watching TV while eating meals is associated with overweight and obesity has to our knowledge not been studied in a large cross European sample of children at this age. However, high rates of overweight and obesity has been shown to be associated with watching TV during meals in a younger sample of children [32]. These issues are important to gain more insight in, since they could be possible targets for early prevention of overweight and obesity in children.

The present study is part of the ENERGY-project (EuropeaN Energy balance Research to prevent excessive weight Gain among Youth) [33]. The aims of this study were to assess: (i) the prevalence of eating breakfast, lunch and dinner, as well as the prevalence of never watching TV during these meals among children, ages 10–12 years in Europe, (ii) the relationship between these behaviors and weight status and (iii) potential country differences and inequalities regarding gender, parental levels of education and ethnicity in these behaviors among children, ages 10–12 years in Europe.

## Materials and methods

The ENERGY- project includes a cross-sectional, school-based survey of anthropometrics and EBRB’s across eight European countries. The design and conceptual framework of the project [33], as well as a description of the cross-sectional survey [34], have been previously published. The present study was conducted according to the guidelines in the Declaration of Helsinki and all procedures involving human subjects were approved by the relevant ethical committees and ministries in each participating country [34].

### Sample and procedure

Seven countries were included in the school-based survey (Belgium, Greece, Hungary, the Netherlands, Norway, Slovenia and Spain), conducted between March and July 2010. Switzerland later joined the research consortium and started their survey in May 2010 and distributed the last questionnaires in December 2010. A national sample frame was used in Greece, Hungary, the Netherlands and Slovenia, while schools from specific regions were sampled in Spain, Belgium, Norway and Switzerland. Pupils in their final years of primary education (aged 10 to 12 years), and one of their parents, were included in the study. The sample size was calculated to detect differences in overweight prevalence between countries. Based on previous cross-European studies a sample of 1000 schoolchildren per country, and one parent (the main caretaker) for each child, were aimed for.

A school recruitment letter was sent to the headmaster of the sampled schools, followed by a personal telephone call. Following the schools agreement, parents received a letter explaining the study purpose and were asked to provide a written consent for their child’s participation in countries where active informed consent (opt in) was required (Belgium, Hungary, Norway, Spain, Greece, Slovenia and Switzerland) or were provided with a form to declare that their child was not to be included in the study in the other countries where medical ethical approval required passive informed consent (opt out). The pupils completed a questionnaire in the classroom in the presence of a trained project worker (approx. 45 minutes). Children participating in the study received a questionnaire to take home for completion by one of their parents. Completed parent questionnaires were brought back to the school in a closed envelope by the children and were collected by the teacher. A total of 199 schools participated, with 7915 children (response rate 60%) and 6512 parents (response rate 55%) completing the questionnaires. The 7915 children constitute the study sample in the present study.

### Measures

All measures were conducted according to standardized protocols across the participating countries [34]. Consistency of questionnaires was further ensured by translating the original questionnaire (developed in English) into each relevant language and then back-translating into English. Only parts of the child questionnaire will be used in the present study, and further information regarding the procedures and training of research staff, and more about other measures [34] are published elsewhere.

### Weight status

Body height and weight were measured by trained research assistants. The children were measured in light clothing without shoes. Body height was measured with a Seca Leicester Portable stadiometer (accuracy of 0.1 cm). Weight was measured with a calibrated electronic scale SECA 861 (accuracy of 0.1 kg). Two readings of each measurement were obtained. If the two readings differed more than 1%, a third measurement was taken. All three measurements were recorded and the outlier was excluded during the data cleaning process and the mean of the two remaining recordings was calculated. BMI was calculated for each child and the definition of weight status (normal weight, overweight, obesity) was based on the International Obesity Task Force criteria [35].

### Personal variables

Gender; “Are you a girl or a boy?” with the response options “girl” and “boy” and ethnicity; “Which language do you most often speak at home?” with the response options: “native language”, “three country specific language options”, “other”, were self-reported in the child questionnaire. The ethnicity variable was dichotomized into: “native” and “non-native”. Parental educational level was assessed in the parent questionnaire. Parents were asked to report their own level of education and that of the other parent/caregiver. Both scores were combined, and dichotomized into low (both parent/caregiver with fewer than 14 years of education) and high (at least one parent/caregiver with 14 years or more of education). In this international dataset this approximately distinguishes families with at least one caregiver who has completed medium or higher vocational, college or university training from other families.

### Eating meals, watching television while eating meals and total TV time

Prevalence of eating meals was assessed by the following questions “Did you eat breakfast/lunch/dinner yesterday?” Each of the three questions had response options: “Yes” and “No”. Tuesdays-Fridays were survey days (children’s weekend meals were not assessed). Test-retest reliability of the three meals recall items was 0.33-0.64 expressed by intraclass correlation coefficient (ICC) in a separate study [36]. A variable was created showing the number of meals eaten yesterday (0–1, 2 or 3 meals). It was only 0.1% of the population that ate 0 meals yesterday, and therefore 0 and 1 meal were collapsed into one category. Prevalence of watching TV while eating meals was assessed with a frequency question: “How often do you watch television during meals?” Meals being: “breakfast”, “lunch” and “dinner” and the response options were: “Always”, “Often”, “Sometimes”, “Not often” or “Never”. The frequency score was dichotomized into never watching TV (i.e. those who “Never” watch TV during breakfast, lunch and dinner) vs. the rest. This rather strict dichotomization was chosen in order to separate those never watching TV during meals from the rest. For the question “How often do you watch television during meals, the test-retest reliability was 0.75-0.77 (ICC) [36]. A variable indicating TV watching at 1, 2 or 3 meals was created. Total TV time was assessed by the question: “About how many hours a day do you usually watch television in your free time?” Weekdays with nine response options ranging from “none at all, 30 minutes/day, 1 hour/day, 1.5 hours/day, 2 hours/day….4 hours/day” and Weekend days with the same nine response options. Total TV time was collapsed into one variable assessing TV time (minutes/week). Test-retest reliability of the total TV time item was 0.68 (ICC) [36].

### Statistics

All data were analyzed using SPSS version 19 (SPSS Inc. Chicago, IL). Chi-square tests were performed to calculate proportions classified as normal weight, overweight and obese, according to gender, ethnicity, level of parental education and country. Proportions of meal consumers and those who never watched TV during breakfast, lunch and dinner were calculated according to weight status, gender, ethnicity, level of parental education and country (Table 1). To determine if the meals variables were associated with each other, and if the never TV at meals variables were associated with each other, we performed Chi-square tests (crude associations between pairs of categorical variables). Crude analyses of associations between eating breakfast and eating lunch revealed a significant relationship, p < 0.001. However, this association was not statistically significant for the relationship between eating lunch and eating dinner. Moreover, there was a statistically significant association between never watching TV at breakfast and never watching TV at lunch, p < 0.001, and also between never TV at lunch and dinner, p < 0.001. Multinomial logistic regression analyses were conducted to calculate odds ratios on the relationship between the respective EBRB’s and weight status in the total sample and for each country separately, adjusting for gender, ethnicity and total TV time (only for never TV at meals variables) as potential confounding factors (Table 2). In addition, adjusting for parental education was performed, but did not alter the results in any major way, and thereby it was decided not to adjust for, because the parental sample is 1400 less than the children sample (Table 2). Binary logistic regression analyses were further performed to assess potential inequalities regarding gender, ethnicity and parental education in terms of not eating meals yesterday and never watching TV during breakfast, lunch and dinner (adjusted for total TV time) compared to eating meals yesterday and those watching TV at meals respectively, in the total sample and also stratified on country (Table 3). As the ENERGY cross-sectional survey used a nested design with children nested within schools, proxies for intra class correlation coefficients (ICC) were calculated based on the proposal by Twisk [37]. All ICCs for the meals and TV at meal variables were considered low (all < 0.08, except for never TV at lunch (ICC = 0.13) and never TV at dinner (ICC = 0.11)) [37]. Therefore, we did not further adjust for the nested design*.* Analyses with subpopulations with fewer than five observations were classified as non-applicable (Na) with regard to the validity of the analysis [38].

**Table 1:** Descriptive analysis of the proportions classified as normal weight, overweight and obese, as well as those who consumed breakfast, lunch and dinner yesterday, and never TV watching at breakfast, lunch and dinner, related to weight status, gender, parental education, ethnicity and country

**Total**	**N**	**Normal weight**	**Overweight**	**Obese**	**Breakfast yesterday**	**Lunch yesterday**	**Dinner yesterday**	**Never TV at breakfast**	**Never TV at lunch**	**Never TV at dinner**
		**%**	**%**	**%**	**%**	**%**	**%**	**%**	**%**	**%**
	**7915**	**77**	**18**	**5**	**85**	**96**	**93**	**55**	**46**	**32**
Normal weight	5953				87	96	95	55	48	34
Overweight	1413				79	96	89	54	40	28
Obese	351				69	95	79	52	33	24
*p*-value					≤0.001	0.20	≤0.001	0.30	≤0.001	≤0.001
Girls	4120	79	17	4	83	96	93	58	58	33
Boys	3795	75	20	5	86	96	93	51	44	32
*p*-value		≤0.001	0.001	0.03	0.001	0.35	0.43	≤0.001	0.002	0.79
Low education	2020	73	21	6	81	96	91	55	43	29
High education	3719	80	17	3	88	97	94	59	50	35
*p*-value		≤0.001	0.002	≤0.001	≤0.001	0.18	≤0.001	0.003	≤0.001	≤0.001
Non-native	617	74	19	6	79	96	92	51	33	28
Native	7175	77	18	4	85	96	93	55	47	33
*p*-value		0.12	0.64	0.02	≤0.001	0.70	0.43	0.03	≤0.001	0.01
Belgium	1008	85	12	3	87	99	99	58	64	42
Greece	1100	59	30	10	76	97	82	60	17	13
Hungary	1022	75	20	5	77	94	92	54	54	28
The Netherlands	959	84	12	4	92	96	98	45	40	42
Norway	1006	86	13	1	93	89	97	41	40	38
Slovenia	1187	73	21	6	73	97	88	60	59	35
Spain	1025	75	22	3	97	100	97	48	32	18
Switzerland	608	86	12	2	85	99	96	84	74	54
*p*-value		≤0.001	≤0.001	≤0.001	≤0.001	≤0.001	≤0.001	≤0.001	≤0.001	≤0.001

**Table 2:** Odds ratios (95% confidence intervals) of being overweight and obese (compared to normal weight) for eating breakfast, lunch and dinner (compared to not eating the respective meals), never TV watching at breakfast lunch and dinner (compared to watching TV at the respective meals) in the total ENERGY-sample and for each country

**Total (N = 7915)**	**Breakfast yesterday**	**Lunch yesterday**	**Dinner yesterday**	**Never TV at breakfast**	**Never TV at lunch**	**Never TV at dinner**
Overweight	*0.6 (0.5-0.7)	0.9 (0.7-1.3)	*0.4 (0.3-0.5)	1.0 (0.9-1.1)	*0.7 (0.7-0.8)	*0.8 (0.7-0.9)
Obese	*0.3 (0.3-0.4)	0.7 (0.4-1.1)	*0.2 (0.1-0.3)	0.9 (0.7-1.1)	*0.6 (0.4-0.7)	*0.6 (0.5-0.8)
**Belgium**						
Overweight	*0.5 (0.3-0.7)	Na	Na	0.8 (0.5-1.2)	0.8 (0.5-1.2)	*0.6 (0.4-1.0)
Obese	0.6 (0.2-1.5)	Na	Na	0.9 (0.4-2.1)	1.1 (0.5-2.4)	0.4 (0.2-1.1)
**Greece**						
Overweight	*0.7 (0.5-0.9)	1.6 (0.6-4.0)	*0.6 (0.4-0.8)	1.3 (1.0-1.7)	0.9 (0.6-1.3)	1.2 (0.8-1.8)
Obese	*0.3 (0.2-0.5)	0.3 (0.1-0.8)	*0.4 (0.2-0.6)	1.0 (0.7-1.5)	0.7 (0.4-1.3)	0.9 (0.5-1.8)
**Hungary**						
Overweight	0.7 (0.5-1.1)	0.9 (0.5-1.7)	*0.5 (0.3-0.9)	1.1 (0.8-1.5)	0.9 (0.7-1.3)	0.9 (0.6-1.3)
Obese	0.9 (0.5-1.8)	Na	*0.3 (0.1-0.6)	0.7 (0.4-1.2)	0.9 (0.5-1.6)	1.7 (0.9-3.2)
**The Netherlands**						
Overweight	0.5 (0.3-1.0)	1.0 (0.4-2.5)	*0.2 (0.0-0.5)	0.5 (0.3-0.9)	*0.6 (0.4-1.0)	0.8 (0.5-1.2)
Obese	*0.2 (0.1-0.5)	0.8 (0.2-3.6)	Na	0.8 (0.3-1.8)	0.7 (0.3-1.7)	0.5 (0.2-1.3)
**Norway**						
Overweight	0.6 (0.3-1.1)	*0.4 (0.3-0.7)	0.7 (0.2-1.8)	1.0 (0.7-1.6)	1.3 (0.8-1.9)	1.3 (0.9-2.0)
Obese	^1^Na	Na	Na	1.4 (0.4-4.2)	1.2 (0.4-3.7)	Na
**Slovenia**						
Overweight	*0.7 (0.5-0.9)	Na	*0.5 (0.3-0.7)	1.1 (0.8-1.5)	1.1 (0.8-1.4)	1.3 (0.4-1.4)
Obese	*0.6 (0.3-0.9)	Na	*0.4 (0.2-0.7)	0.6 (0.4-1.1)	*0.5 (0.3-0.9)	0.8 (0.4-1.4)
**Spain**						
Overweight	0.7 (0.3-1.6)	Na	0.8 (0.3-2.0)	0.7 (0.5-1.0)	0.8 (0.6-1.1)	0.8 (0.5-1.3)
Obese	Na	Na	Na	0.6 (0.3-1.4)	Na	Na
**Switzerland**						
Overweight	0.6 (0.3-1.1)	Na	0.4 (0.1-1.2)	0.7 (0.4-1.4)	0.7 (0.4-1.4)	1.0 (0.6-1.7)
Obese	Na	Na	Na	Na	Na	1.4 (0.4-4.3)

* p ≤ 0.05, reference category: normal weight, adjusted for gender, ethnicity and total TV time (only TV at meals variables) as potential confounding factors.

^1^Na: Non applicable due to fewer than 5 observations in subpopulations.

**Table 3:** Odds ratios (95% confidence intervals) of eating breakfast, lunch and dinner yesterday (compared to not eating the respective meals), never TV watching at breakfast, lunch and dinner (compared to watching TV at the respective meals, ¥) related to gender, ethnicity and parental education in the total ENERGY-sample and for each country

**Total (N = 7915)**	**Breakfast yesterday**	**Lunch yesterday**	**Dinner yesterday**	**Never TV at breakfast**	**Never TV at lunch**	**Never TV at dinner**
Gender (boys vs. girls)	*1.2 (1.1-1.4)	1.0 (0.7-1.3)	1.0 (0.8-1.3)	*0.8 (0.7-0.9)	0.9 (0.8-1.0)	1.0 (0.9-1.1)
Ethnicity (native vs. non-native	*1.4 (1.0-1.8)	0.8 (0.4-1.5)	1.0 (0.7-1.5)	1.0 (0.8-1.2)	*1.4 (1.1-1.8)	1.0 (0.8-1.3)
Education (high vs. low)	*1.7 (1.5-2.0)	1.2 (0.9-1.7)	*1.5 (1.2-1.4)	1.1 (1.0-1.2)	*1.2 (1.0-1.3)	*1.2 (1.1-1.4)
**Belgium**						
Gender (boys vs. girls)	1.6 (1.0-2.6)	2.3 (0.4-12.0)	0.8 (0.2-3.1)	*0.7 (0.5-1.0)	*0.7 (0.5-1.0)	1.0 (0.7-1.3)
Ethnicity (native vs. non-native	1.1 (0.4-2.7)	Na	Na	1.2 (0.6-2.3)	*2.0 (1.0-3.9)	1.3 (0.6-2.5)
Education (high vs. low)	*2.0 (1.1-3.5)	Na	Na	1.4 (0.9-2.3)	1.3 (0.8-2.1)	1.2 (0.8-1.9)
**Greece**						
Gender (boys vs. girls)	1.1 (0.8-1.5)	0.6 (0.2-1.3)	0.9 (0.6-1.2)	*0.7 (0.5-0.9)	0.9 (0.7-1.4)	1.2 (0.8-1.8)
Ethnicity (native vs. non-native	0.9 (0.5-1.6)	*6.8 (2.7-17.2)	0.8 (0.4-1.6)	1.4 (0.8-2.3)	0.9 (0.5-1.8)	1.2 (0.5-2.7)
Education (high vs. low)	1.4 (1.0-1.9)	0.7 (0.3-1.6)	1.1 (0.8-1.6)	1.1 (0.8-1.5)	1.0 (0.7-1.5)	*1.5 (1.0-2.3)
**Hungary**						
Gender (boys vs. girls)	1.6 (1.1-2.2)	1.7 (0.9-3.4)	1.1 (0.6-1.9)	*0.7 (0.5-1.0)	1.0 (0.7-1.3)	0.9 (0.6-1.3)
Ethnicity (native vs. non-native	2.1 (0.8-5.8)	Na	Na	0.8 (0.3-2.3)	1.2 (0.4-3.5)	0.5 (0.2-1.5)
Education (high vs. low)	0.8 (0.5-1.1)	0.7 (0.4-1.3)	1.0 (0.6-1.6)	*1.4 (1.1-2.0)	*1.5 (1.1-2.0)	1.0 (0.7-1.5)
**The Netherlands**						
Gender (boys vs. girls)	0.4 (0.1-1.5)	0.7 (0.2-2.4)	Na	1.3 (0.8-2.0)	1.2 (0.8-1.9)	1.0 (0.6-1.5)
Ethnicity (native vs. non-native	^1^Na	Na	Na	3.8 (0.7-20.1)	3.8 (0.7-19.1)	2.1 (0.5-8.5)
Education (high vs. low)	Na	2.8 (0.8-9.0)	Na	*1.9 (1.0-3.4)	1.2 (0.7-2.1)	1.0 (0.6-1.8)
**Norway**						
Gender (boys vs. girls)	1.0 (0.5-1.9)	0.9 (0.5-1.5)	1.0 (0.4-2.3)	0.9 (0.7-1.3)	1.1 (0.8-1.5)	0.8 (0.6-1.1)
Ethnicity (native vs. non-native	2.1 (0.6-7.5)	Na	Na	*0.4 (0.1-1.0)	1.0 (0.4-2.5)	0.8 (0.3-1.9)
Education (high vs. low)	*3.1 (1.6-5.9)	1.3 (0.8-2.4)	1.5 (0.6-3.9)	*1.9 (1.3-2.8)	1.0 (0.7-1.4)	1.4 (1.0-2.0)
**Slovenia**						
Gender (boys vs. girls)	1.2 (0.9-1.7)	0.6 (0.3-1.4)	1.3 (0.9-2.0)	0.9 (0.7-1.2)	0.8 (0.6-1.1)	1.1 (0.8-1.5)
Ethnicity (native vs. non-native	1.4 (0.8-2.4)	Na	0.7 (0.3-1.7)	1.5 (0.9-2.7)	*4.5 (2.3-8.6)	1.6 (0.8-3.0)
Education (high vs. low)	*1.4 (1.0-1.9)	2.0 (0.9-4.6)	1.1 (0.7-1.6)	*1.5 (1.1-2.1)	*1.4 (1.1-1.9)	1.2 (0.9-1.6)
**Spain**						
Gender (boys vs. girls)	0.9 (0.4-1.9)	Na	1.1 (0.5-2.7)	*0.7 (0.5-0.9)	0.7 (0.6-1.0)	1.0 (0.7-1.5)
Ethnicity (native vs. non-native	Na	Na	Na	1.2 (0.5-3.0)	1.2 (0.4-3.3)	4.4 (0.6-34.0)
Education (high vs. low)	1.0 (0.3-2.6)	Na	Na	1.2 (0.9-1.7)	*1.6 (1.1-2.4)	*1.7 (1.0-2.8)
**Switzerland**						
Gender (boys vs. girls)	0.9 (0.6-1.5)	Na	0.7 (0.3-1.7)	0.8 (0.4-1.3)	0.9 (0.6-1.4)	0.8 (0.6-1.2)
Ethnicity (native vs. non-native	1.1 (0.6-2.0)	Na	1.8 (0.7-4.6)	*2.2 (1.2-3.8)	*3.0 (1.9-5.0)	1.3 (0.8-2.1)
Education (high vs. low)	1.4 (0.9-2.4)	Na	1.1 (0.5-2.8)	1.5 (0.9-2.7)	1.3 (0.8-2.2)	1.3 (0.9-1.9)

*p ≤ 0.05.

^1^Na: Non-applicable due to fewer than 5 observations in subpopulations.

¥ adjusted for total TV time.

## Results

The study sample included 7915 children; mean age 11.5 years; 52% girls, 65% high parental education and 92% native ethnicity. Further, 77%, 18% and 5% were categorized as normal weight, overweight and obese, respectively (Table 1). Children reporting eating breakfast, lunch, and dinner yesterday were 85%, 96%, and 93% respectively. Children reporting to never watch TV while eating breakfast, lunch and dinner were 55%, 46% and 32% respectively (Table 1).

Adjusting for gender and ethnicity as potential confounders, the children who ate breakfast (OR = 0.6 (95% CI 0.5-0.7)) and dinner (0.4 (95% CI 0.3-0.5)), had lower odds of being overweight compared to those who did not eat the respective meals (Table 2). The children who ate breakfast (0.3 (95% CI 0.3-0.4)) and dinner (0.2 (95% CI 0.1-0.3)) had lower odds of being obese, compared to those who did not eat the respective meals. Significant relationships between meals and overweight/obesity were also observed within some of the countries, all in the same direction as the results computed for the total study sample (Table 2). Of the total sample, 76% ate all three meals yesterday, 19% had two meals and 3% had 0 or 1 meal. Adjusting for gender and ethnicity as potential confounders, the children who ate all three meals had lower odds of being overweight than those who ate 0–1 meal (0.4 (95% CI 0.3-0.6, p < 0.001)) and two meals (0.5 (95% CI 0.5-0.6, p < 0.001)), and the children who ate all three meals also had lower odds of being obese compared to those who ate 0–1 meal (0.1 (95% CI 0.1-0.2, p < 0.001)) and two meals (0.3 (95% CI 0.3-0.4, p < 0.001)).

Adjusting for gender, ethnicity and total TV time as potential confounders, the children who never watched TV at lunch (0.7 (95% CI 0.7-0.8)) and dinner (0.8 (95% CI 0.7-0.9)), had lower odds of being overweight compared to those who did (Table 2). The children who never watched TV at lunch (0.6 (95% CI 0.4-0.7)) and dinner (0.6 (95% CI 0.5-0.8)), had lower odds of being obese compared to those who did (Table 2). In the country-stratified analyses, few significant relationships were found, although the ORs were in the similar direction across the different countries (Table 2). Of the total sample, 21% never watched TV at meals, 19% watched TV at one meal, 28% watched TV at two meals and 28% watched TV at three meals. The children who never watched TV at meals did not have significantly lower odds of being overweight or obese than the children who watched TV at one meal (data not shown). Adjusting for gender, ethnicity and total TV time as potential confounders, the children who never watched TV at meals had lower odds of being overweight compared to those who watched TV at two meals (0.7 (95% CI 0.6-0.8, p < 0.001)) and three meals (0.8 (95% CI 0.6-0.9, p = 0.01)), and the children who never watched TV at meals had lower odds of being obese compared to those who watched TV at two meals (0.5 (95% CI 0.4-0.8, p = 0.001)) and three meals (0.6 (95% CI 0.4-0.8, p = 0.003)).

Regarding potential socio-demographic determinants, boys were more likely to have had breakfast (1.2 (95% CI 1.1-1.4)) compared to girls in the total sample (Table 3). Children of native ethnicity of the country of administration had higher odds for eating breakfast (1.4 (95% CI 1.0-1.8)) compared to non-natives (Table 3). Children of highly educated parents were more likely to have had breakfast (1.7 (95% CI 1.5-2.0)) and dinner (1.5 (95% CI 1.2-1.4)) compared to children of lower educated parents (Table 3). These differences were similar, but most often not statistically significant in the country-stratified analyses (Table 3).

Boys were less likely to report to never watch TV while eating breakfast (0.8 (95% CI 0.7-0.9)) compared to girls in the total sample (Table 3). Children from native ethnicity were more likely to never watch TV at lunch (1.4 (95% CI 1.1-1.8)) compared to non-natives (Table 3), and children of higher educated parents were more likely to never watch TV at lunch (1.2 (95% CI 1.0-1.3)) and dinner (1.2 (95% CI 1.1-1.4)) compared to children of lower educated parents (Table 3). Some of these associations were also found to be significant in the country-stratified analyses (Table 3).

## Discussion

The present study indicates that both eating meals and never watching TV while eating meals were associated with lower odds of overweight and obesity. Children who reported to have eaten breakfast and dinner on the day before questionnaire administration, were significantly less likely to be overweight or obese than those who did not eat the respective meals, and children who reported to never watch TV at lunch and dinner were significantly less likely to be overweight or obese than those who did. Children who ate breakfast were also more likely to eat lunch, and therefore variables of meals combined were assessed showing that children eating all three meals were less likely to be overweight or obese than those who ate 0–2 meals. TV at meals variables were also associated with each other, and the children who never watched TV at meals were less likely to be overweight or obese than those who watched TV at two or three meals.

A systematic review on European children and adolescents suggest that eating breakfast is associated with a reduced risk of becoming overweight or obese and a reduction in BMI [10], and previous cross-sectional studies [39,40] and a longitudinal study [9] observed that children and adolescents skipping breakfast are more likely to have higher BMI compared to regular breakfast consumers. This relationship was also found in our data. There were some country differences regarding eating meals on the day before questionnaire administration in our study. In Greece, Hungary and Slovenia less children reported eating breakfast and dinner than children from the other countries in the study. The prevalence of overweight and obesity of children from these three countries are also among the highest in the study sample. The strongest predictor of being overweight was skipping dinner and eating 0–1 meal (vs. 3 meals). However, the 95% confidence interval of these estimates overlaps with the 95% CI of the skipping breakfast variable so we cannot conclude that these variables are statistically significantly different from one another. The strongest predictor of being obese was eating 0–1 meal (vs. 3 meals), which again has an overlapping CI with the skipping dinner variable. Koletzko et al. found a significant reduction of obesity risk with increasing number of meals [13]. Eating meals together with your family may reduce meal skipping [41], and children who eat more meals with their families have been reported to consume more healthful diets [42].

Dubois et al. found that children who once daily or more reported to eat while watching TV had significantly higher mean BMI in comparison to children who reported fewer TV watching events at meals [29]. In a large cohort of Canadian children (5th grade), eating while watching TV was positively associated with overweight and poor nutrition [30]. Watching TV during a meal has been reported to contribute to increased energy intake and could thereby be associated with increased BMI [43-45]. Another reason why TV watching during meals might affect obesity status might be that TV watching may be associated with more ‘mindless’ eating, and may thus increase the amount of foods and thus the amount of calories consumed [46]. Children are also exposed to advertising of energy-rich and nutrient poor food and beverages while watching TV, and Lobstein et al. [28] found evidence for a link between advertising to children and the risk of overweight among children in the USA, Australia and eight European countries. A significant association was found between children’s overweight and the numbers of advertisements per hour on children’s TV, especially those advertisements that encourage the consumption of energy-dense, micronutrient-poor foods suggesting that the quantity of advertising on TV for children appears to be related to the prevalence of excess body weight [28]. The strongest predictor of being overweight among the TV at meals related variables was watching TV at lunch and watching TV at two meals (vs. never TV at meals). However, there was an overlap of the confidence intervals with TV at dinner and TV at three meals variables, so we cannot conclude that these estimates are statistically significantly different from each other. The strongest predictor of being obese was TV watching at two meals, again with overlapping CI’s. Promotion of family meals may be a way of avoiding eating in front of the television. The association between total TV time and never TV watching at meals was tested, and it was found that the negative relationship between never watching TV at lunch and dinner (but not breakfast) and overweight or obesity still remained significant. This indicates that TV at meals or not may play an independent role on weight status in this study, and are not likely to be explained by the fact that these children just watch TV a lot.

As previously described in the literature [7,47], the present study also observed significant associations between gender, ethnicity and parental education and meal pattern. Being a boy, native and having parents where at least one had more than 14 years of education were associated with higher odds for eating breakfast. Likelihood of eating dinner was associated with higher education of parents. Merten et al. [48] reported that low-income youth from disadvantaged communities were more likely to skip breakfast. We found that children with at least one parent with higher education were more likely to never watch TV at lunch and dinner. Dubois et al. [29] found that a larger proportion of children from mothers with lower socio-economic status watched more TV and also watched more TV frequently during meals and snacks. Coon et al. [49] reported that a greater proportion of children who ate while watching TV came from families with low family income and low socio-economic status. Our findings that natives are more likely to never eat lunch in front of TV are in line with Dubois et al. who found that children with an immigrant mother, as opposed to a non-immigrant mother, were significantly more likely to eat breakfast and snacks in front of the TV every day [29].

### Strengths and limitations of the study

Strengths of the present study include the large multinational sample from different regions across Europe, the application of a standardized data collection protocol across the different countries and that weight and height was objectively measured. The self-reported measures were test-retested and validated in a separate study [36]. The study also has limitations. There were some differences in response rates at school and at the pupil level, and this may have reduced the external validity of the findings. Lower ICCs are to be expected regarding 24 h recall questions, because children will have larger variety in activities engaged in yesterday compared to on a usual day. There is no frequency data available for eating lunch and dinner, but there is good correlation between the 24 h recall question and a frequency question (FFQ) on eating breakfast. For the children who did not eat breakfast yesterday, the mean value on the FFQ measure was 1.4 days/week with breakfast, while for the children who ate breakfast yesterday, the mean value was eating breakfast 4.6 days/week (*t*-test, p-value ≤ 0.001). Because of the cross-sectional design of the study, we cannot draw any inferences about causality, i.e. if skipping meals leads to higher BMI or if children that are aware of their overweight status tend to skip meals. There are probably many aspects of socioeconomic differences i.e. family income that are not covered by the variables included in the present study. One country (Switzerland) had a lower inclusion of children (577) compared to the other seven countries (i.e. approximately 1000) making the comparison possibly somewhat biased.

## Conclusions

This study indicates that the likelihood of being overweight was lower for children who ate breakfast and dinner on the day before questionnaire administration, compared to those who did not eat the respective meals. The odds ratio of being overweight was lower for children who reported to never watch TV at lunch and dinner compared to those who did. Since this study is based on cross-sectional data, longitudinal and interventions studies are needed to gain a better understanding of causality.

## Abbreviations

EBRB: Energy-balance related behavior; TV: Television; BMI: Body mass index.

## Competing interests

The authors declare that they have no competing interests.

## Authors’ contribution

FVN and EB designed the present study. JB conceived the ENERGY project. JB, EB and YM designed the experiments. EB, FNV, OA, LM, NJ, EK, LAM, AD and JB contributed to or supervised the national data collection procedures. FNV conducted the data analyses under supervision of EB. FNV drafted the manuscript. All authors provided feedback on drafts and approved the final manuscript.
